# High-throughput sequencing: a failure mode analysis

**DOI:** 10.1186/1471-2164-6-2

**Published:** 2005-01-04

**Authors:** George S Yang, Jeffery M Stott, Duane Smailus, Sarah A Barber, Miruna Balasundaram, Marco A Marra, Robert A Holt

**Affiliations:** 1 Canada's Michael Smith Genome Sciences Centre, BC Cancer Research Centre, Suite 100, 570 West 7^th ^Avenue, Vancouver, B.C., Canada

## Abstract

**Background:**

Basic manufacturing principles are becoming increasingly important in high-throughput sequencing facilities where there is a constant drive to increase quality, increase efficiency, and decrease operating costs. While high-throughput centres report failure rates typically on the order of 10%, the causes of sporadic sequencing failures are seldom analyzed in detail and have not, in the past, been formally reported.

**Results:**

Here we report the results of a failure mode analysis of our production sequencing facility based on detailed evaluation of 9,216 ESTs generated from two cDNA libraries. Two categories of failures are described; process-related failures (failures due to equipment or sample handling) and template-related failures (failures that are revealed by close inspection of electropherograms and are likely due to properties of the template DNA sequence itself).

**Conclusions:**

Preventative action based on a detailed understanding of failure modes is likely to improve the performance of other production sequencing pipelines.

## Background

In the past decade, the demand for DNA sequence data has driven the transformation of sequencing from a research activity into a manufacturing process. High-throughput sequencing facilities are focused on establishing automated procedures that maintain long read length and high overall success rates. It is neither practical nor economical to test each and every DNA template before sequencing [1]. Sequencing centres, therefore, monitor sequencing success on a larger scale referencing overall pass rates and average read lengths, typically in terms of Phred 20 bases [2]. The percentage of "sporadic sequence dropouts" or failed reads that inevitably occur within a pool of high quality data is often overlooked and rarely examined. Failed reads can be a result of numerous variables ranging from pipeline methodology employed to the nature of samples being sequenced. A Failure Mode Analysis (FMA) strategy was developed to determine the likely causes of sporadic unsuccessful sequence reads. We systematically examine these failed reads in the context of a high-throughput sequencing pipeline to establish the mode and frequency of each type of failure. The standard production pipeline at Canada's Michael Smith Genome Sciences Centre (BCCRC, British Columbia Cancer Agency, Vancouver, Canada) has a capacity to generate over 3.6 million reads per year. As of December 8, 2004, we have generated 1,263,904,347 Q_20 _bases using our 384-well culturing, DNA preparation, and cycle sequencing procedures. The average Q_20 _read length of data generated in the past 12 months (December 2003 to December 2004) from various library types and vector systems is 751 bases. The present study was undertaken to provide insight into the causes of sequencing failures and possible corrective actions. Although our pipeline uses exclusively ABI 3700 and 3730XL automated sequencers, these results should be applicable, in principle, to the improvement of other high throughput sequencing platforms.

## Results

**Figure 1:**
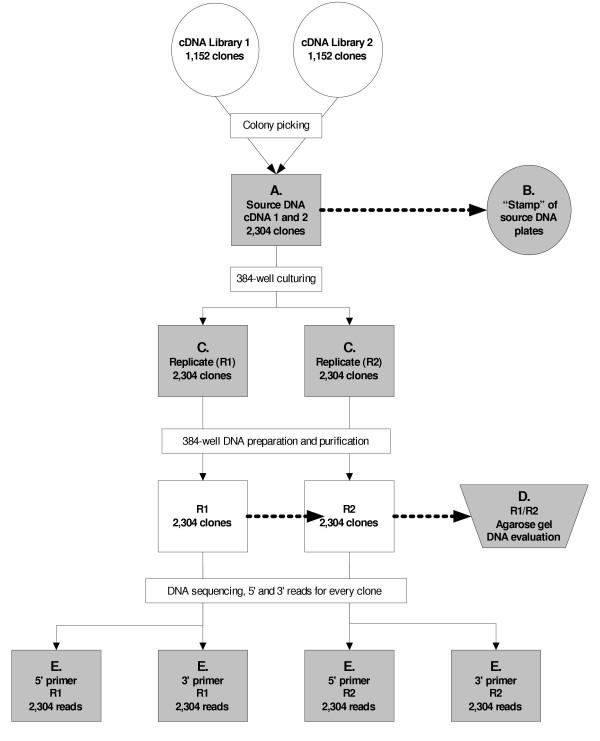
**Process pipeline. **Observational checks within the pipeline are shaded in grey. Absence of bacterial colonies, no-grows, and unusual observations are recorded on logsheets then entered into the FMA database. A. Verification of the colony picking procedure to ensure that all original clones are accounted for in the source microtiter plates. B. To further confirm A, we stamp a replicate of each microtiter plate containing transformed bacterial cultures onto agar plates. The resulting pattern of colonies is examined to determine presence or loss of DNA in each well of the source microtiter plate. C. Every 384-well culture plate is visually examined for presence of DNA after bacterial DNA culturing. D. Agarose gel electrophoresis is used to evaluate presence and quality of prepared and purified template DNA. E. During sequencing reactions, all volume additions are visually verified and manually adjusted using a single channel pipettor where necessary.

**Figure 2:**
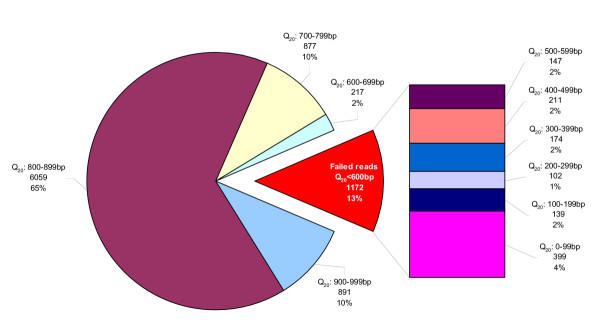
**Average read length breakdown **Distribution of read length (Q_20 _bases) for full data set of 9,216 reads. Results were divided into 100 bp bins, failed reads (Q^20^<600 bp) make up 13% (1,172 reads) of overall reads. Reads with Q^20^<100 bp make up the largest proportion of failed reads but contributes to only 4% of overall data set.

**Figure 3:**
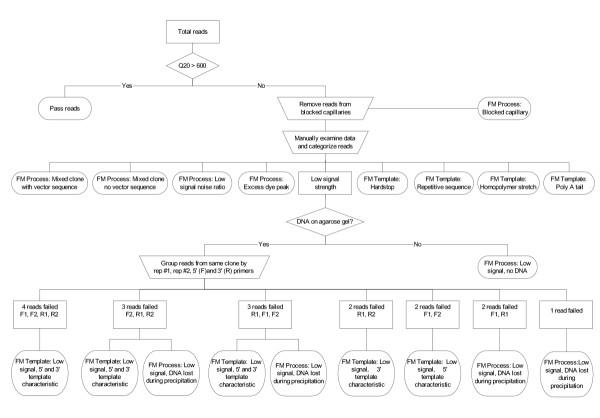
**Analysis pipeline **Analysis methodology used to determine failure modes. FM= Failure Mode.

We generated 9,216 reads from 2,304 clones selected randomly from two cDNA libraries. For each of the two libraries, 1,152 bacterial colonies containing cDNA inserts were picked and arrayed into 384-well microtiter plates (Figure 1). To verify loss of DNA due to handling or equipment mishaps (i.e. clogged capillary or tip), each microtiter plate was cultured in duplicate and replicates were processed using the same instrument model but on different physical units where available. A resulting 4,608 reads were generated for the 5' end using the M13Reverse (5'-CAGGAAACAGCTATGAC-3') primer and 4,608 reads were generated from the 3' end using the M13 Forward (5'-TGTAAAACGACGGCCAGT-3') primer. The average Q_20 _read length for the entire data set was 771 bases, average pass rate was 87% which was calculated as a percentage of sequencing reactions yielding a minimum of 600 Phred 20 bases. Figure 2 illustrates a break down of Q_20 _read lengths from the full data set. The analysis methodology employed to determine the failure mode of each trace is outlined in Figure 3. 1,172 reads (13%) represent the failed portion of the data set (Q_20 _< 600) for further analysis to determine failure mode. The electropherograms from the 1,172 failed reads were evaluated and subsequently categorized into failure mode categories. 64 of these reads were yielded from sequencer capillaries that were clogged and therefore were removed from further analysis and categorized into the "Blocked capillary" failure mode. The remaining 1,108 traces were further classified into nine additional failure mode categories including Low signal strength, Mixed clone with vector sequence, Mixed clone- no vector sequence, Low signal to noise ratio, Excess dye peaks, Hardstop, Repetitive sequence, Homopolymer stretch, and Poly A tail. Results and trace characteristics used to classify each read are as described in Table 1. Eight of the classifications described in Table 1, except "Low signal strength", are final failure mode categories and contain 74% of the total reads.

**Table 1:** Failure mode categories Failed wells were distributed into each category based on observational data taken during sequencing pipeline procedures and manual evaluation of electropherogram traces.

Failure Mode	Trace characteristic	No. of sequencing reactions	Percent of all failed wells (Q_20 _< 600)
Blocked capillary	Noisy or no data with a low signal intensity value (<100). Verified with capillary control results.	64	5.5
Low signal strength*	Noisy or no data with a low signal intensity value (<100) that is very close to or falls below the instruments detectable limit.	310	26.5
Mixed clone w/ vector sequence	Clean vector sequence followed by noisy data immediately after the cloning site.	137	11.7
Mixed clone, no vector sequence	Noisy data throughout the trace with sufficient signal intensity.	27	2.3
Low signal to noise ratio	Discernable sequence peaks with strong intensity background noise.	22	1.9
Excess Dye peaks	Large dye front usually followed by noisy data.	10	0.9
Hardstop	Abrupt end to good sequence.	2	0.2
Repetitive Sequence	Long stretch of repetitive DNA sequence that is followed by slippage in sequence or noisy data.	17	1.5
Homopolymer stretch	Long stretch of a single nucleotide followed by slippage in sequence or noisy data.	99	8.4
Poly A Tail	Stretch of Ts (template A) followed by slippage in sequence or noisy data.	484	41.3
Total		1172	100.0

* Preliminary failure mode, further broken down to final failure modes in Table 2.

**Figure 4:**
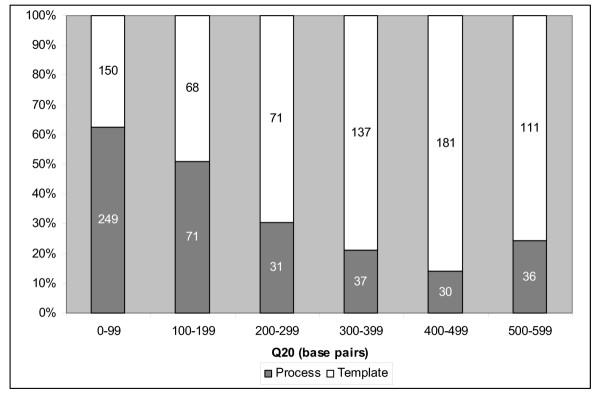
**Distribution of overall process vs template failed reads **Percentage of process versus template-related failures for each 100 bp Q_20 _read length bin. Numbers in each column represent the number of reads in each bin category. Process-related failure modes are more prevalent in lower average Q_20 _read lengths. Template-related failure modes are more prevalent in higher average Q_20 _read lengths.

**Figure 5:**
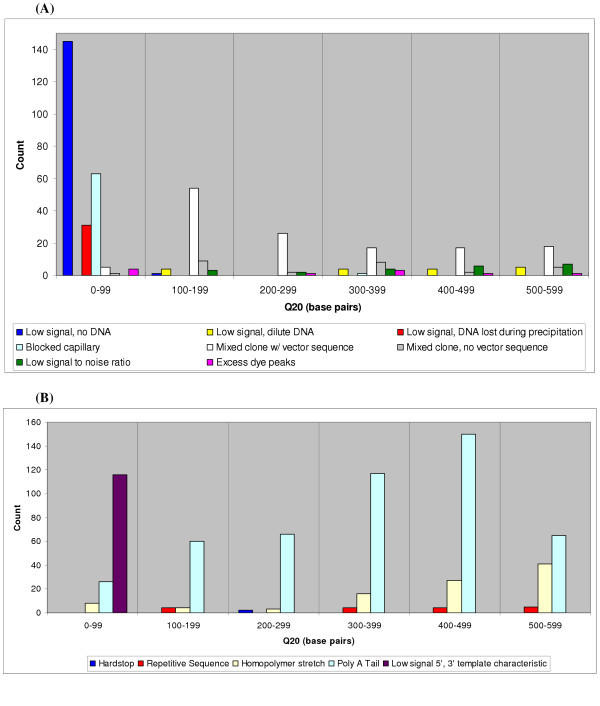
**Process failure mode distribution **(A) Process-related failure mode distribution. Most of the process-related failed reads have a Q_20 _< 100 with the primary mode of failure being "Low signal, no DNA," or no template DNA in the sequencing reaction (confirmed by agarose gel electrophoresis). (B) Template-related failure mode distribution. Most of the template-related failed reads are a result of "Poly A Tail," or 3' poly A stretch that leads to slippage in sequence data. Reads with a "Low signal 5', 3' template characteristic" failure mode contribute to the largest proportion of data with read lengths Q_20 _< 100.

We identified various failure mode trends by examining process versus template-related failures. Figure 4 shows 62.4% of reads with Q_20_<100 bp fail as a result of process-related problems while 75.5% of reads in the Q_20_: 500–599 bp bin fail due to template-related characteristics. The proportion of failed reads due to process-related problems are more abundant in traces with lower average read lengths while the opposite trend showing template-related failures increasing with increased average read length is true. A breakdown of failure modes for each 100 bp Q_20 _bin is shown for process-related failed reads in Figure 5A and template-related failures in Figure 5B.

The most common mode of failure due to process was "low signal, no DNA", there were 146 reads in this category, where no template DNA was present in the sequencing reactions. This was confirmed by agarose gel electrophoresis. 145 of the reads resulted in read lengths less than 100 bp. From these 146 reads, 128 original clones failed to grow in the source glycerol stock microtiter plates. The remaining 18 clones were lost during the DNA preparation/purification procedure. Mixed clone reads containing vector sequence was the second most prevalent process-related failure mode. The 137 reads in this category were distributed broadly across the Q_20_: 100–599 bp range. The more successful reads yielded electropherograms with significantly stronger signal strength from one reaction product compared to the other.

The most common failure mode resulting from template-related characteristics was "Poly A tail" or failed reads with attenuated sequence due to poly A tails, there were 484 reads in this category. The distribution of these 484 reads is skewed towards the Q_20_>300 bp bins. Failures due to "Low signal, 5', 3' template characteristics" also contribute to a significant number of template-related failures. These 116 reads were Q_20_<100 bp and make up the majority failure mode within that Q_20 _bin.

**Figure 6:**
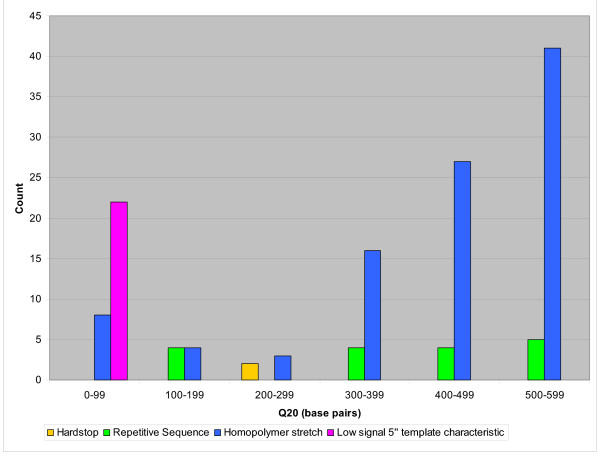
**Template failure mode distribution **Template-related failure mode distribution excluding reads failing due to poly A tail and 3' template-related attributes. A majority of failed reads are a result of "Homopolymer" or single nucleotide repeat sequence leading to slippage in sequence data, these failed reads are skewed towards read lengths Q20>300.

Our sample set was made up of cDNA clones and therefore contain some 3' template biases inherent in the sequencing of cDNAs. In a best effort to obtain a distribution representing randomly generated end sequence from various library types, we remove all failed reads resulting from 3' template attributes. The two failure modes targeted for removal are "Poly A tail" and the 3' reads within the "Low signal, 5', 3' template characteristics" category. 484 and 94 reads were removed from each respective category representing 49.3% of all failed reads. The resulting template-related failure mode distribution is represented in Figure 6. The process-related distribution of failed reads (Figure 5A) remains the same. Failed reads due to homopolymer stretches other than those resulting from poly A tails, make up the prominent failure mode within this new template-related failure distribution with 99 reads. The distribution of these reads is weighted towards Q_20_>300.

We further analyzed the 310 reads (26.5% of failed) in the preliminary "Low signal strength" category in Table 1 to more finely determine each failure mode (Figure 2, Table 2). We removed the 146 reads and classified them as process-related, "low signal, no DNA" failures as described above. For the remaining 164 reads, DNA was found to be present but there was no evidence from gel images for excess DNA. It is therefore unlikely that the low signal failures were due to overloading of capillaries with excess carryover template DNA.

**Table 2:** Breakdown of "Low signal strength" reads Reads from the preliminary "Low signal strength" failure mode category (310 reads) are further categorized into finer failure mode classifications. Failed reads from each unique clone are grouped together where possible (excluding reads that do not confirm presence of DNA on evaluation agarose gel) to determine mode of failure.

Groupings of failed reads	No. of occurrences	Process associated failures (No. of reads)	Template associated failures (No. of reads)	Failure mode
F1/F2/R1/R2	4	0	16	Low signal, 5' and 3' template characteristic
F1/F2/R1	1	1	2	1. Low signal, DNA lost during precipitation 2. Low signal, 5' template characteristic
F2/R1/R2	1	1	2	1. Low signal, DNA lost during precipitation 2. Low signal, 3' template characteristic
R1/R2	43	0	86	Low signal, 3' template characteristic
F1/F2	5	0	10	Low signal, 5' template characteristic
F1/R1	1	2	0	Low signal, DNA lost during precipitation
No available clone pairing (singleton)	44	44	0	Low signal, DNA lost during precipitation
No DNA in agarose gel	146	146	0	Low signal, no DNA

The 164 reads that appear to have sufficient template DNA are grouped by source clone for further analysis. Four possible reads may have failed for each unique clone: 5' replicate #1 (F1), 3' replicate #1 (R1), 5' replicate #2 (F2), and 3' replicate #2 (R2). There were 49 occurrences where two reads failed from one source clone, 2 instances where three reads from the same clone failed and 4 occurrences where all four reads failed from the same source clone. These results are shown in Table 2 and Table 3 summarizes the overall breakdown of 310 reads in the Low Signal Strength category. A binomial test of our data set indicates that the likelihood of 2, 3, or 4 reads failing from the same source clone is 1.9 × 10^-3^, 2.2 × 10^-5^, and 9.7 × 10^-8^, respectively. Thus, it is unlikely that more than one read failed per clone by chance.

**Table 3:** Summary of "Low signal Strength" reads Summarizing results of the 310 "Low signal strength" reads in Table 2.

Low Signal Strength Failure Mode	No. of reads
Low signal, no DNA	146
Low signal, 5', 3' template characteristic	116
Low signal, DNA lost during precipitation	48
Total	310

Groupings with two failed reads from the same end of the template, "F1/F2" and "R1/R2" showed trace data with noisy background or no signal trace therefore there was insufficient information to determine actual template characteristic leading to the problematic results. With the low probability of seeing two failed traces from one unique clone, we conclude that these failures are most likely a result of template DNA with 5' or 3' ends that are problematic for sequencing. Possibilities include secondary structure, mutated priming sites, long poly A tail, or other homopolymer stretches near the priming sites on the template.

The "F1/R1" grouping contained failed reads from both ends of the same replicate but successful end reads from the second replicate. This would indicate that there were no clone related attributes on either end of the template to prevent good sequence thus the failed reads were assessed a process-related failure due to loss of DNA during the precipitation process.

The "F1/F2/R1/R2" grouping indicates that no passing sequence data were obtained from 5' or 3' ends of both replicates from the original source clone, all 16 reads showed presence of DNA at each observational check in the process pipeline. The probability that 4 reaction products from a single clone were lost during precipitation is extremely improbable and it is more likely that the original clones had attributes that prevented successful sequencing from either ends. We therefore assign 5' and 3' template-related failure modes for the 16 reads in this grouping.

The final groupings of "F1/F2/R1" and "F2/R1/R2" contain 6 reads in total. Each include failed reads from both ends of one replicate and a single failed read from the second replicate. We maintain the same logic for our failure mode evaluation by assessing both a template-related failure to the 2 failed reads from the same replicate and a process-related loss of DNA during precipitation failure for the single read from the second replicate.

There were 44 reads in the singleton category where only a single read (F1, F2, R1, or R2) failed from each clone. These failures cannot be attributed to the template as three other reads were successfully sequenced from the same source clone. Failure mode was therefore likely to be process related and due to loss of DNA during the precipitation wash steps.

## Discussion

Having established a stringent pass criteria of 600 Phred 20 bases or greater, we observed a broad range of failure modes distributed among the failed reads. Reads less than 100 bp did not yield useable data and were usually a result of process-related failure modes. Process-related mishaps in the pipeline would include missed colony picks, blocked capillaries on sequencers, faulty tips on automated liquid handling devices, or lost DNA template during precipitation. The sequences generated have low average read lengths, or in cases where DNA is totally lost, zero read length. 88% of reads within the largest "process" related failure mode category, "low signal, no DNA", were a result of failed cultures at the beginning of the process pipeline. The absence of DNA was verified early on by failed growth in the source microtiter plates and further confirmed by failed growth in the stamped replicate agar plate. Failures are perhaps due to missed bacterial colony picks and adjustments such as recalibration or refining the morphology criteria of the colony picker should help. The second most prevalent failure mode in the "process related" category, "Mixed clone with vector sequence" (30% of reads in this classification), is likely a result of two side by side colonies being picked into the same well or through contamination originating from the use of automated liquid handlers. These problems might be alleviated by increasing the stringency of the proximity criteria on the colony picking device to ensure sufficient distance exists between two bacterial colonies before they are chosen for picking. As we use new tips on the instruments daily, increasing the length or number of wash cycles on the automated liquid handlers between volume transfers may help in reducing carry over contamination from one plate to another. This will need further investigation. Cross contamination between adjacent wells can also arise with the mishandling of plates, or when tips are plunged too deeply into the wells, displacing the volume into adjacent wells. Adjusting the liquid handler by reducing the depth and speed of tips entering each well may help prevent the occurrences of cross contamination within a plate. Despite the presence of mixed clones within one well, traces often yielded discernable sequence with read lengths broadly distributed up to the Q_20_: 500–599 bp bin. This is likely the result of a presence in greater amount of one reaction product over the second leading to proportionately stronger signal strength.

Template-related failures often still yielded reads of several hundred base pairs. Template characteristics that might contribute to failed or truncated reads could be poly A tails, homopolymer stretches, or other highly repetitive regions. Problems in sequencing these types of regions are well cited in the literature and are dependent on chemistry and methodology used in sequencing. These problems are best avoided by using an anchored primer for first strand synthesis of cDNA as the first step in library construction. "Poly A tail" was the single largest cause of failed sequence in both the template-related failure category as well as the entire data set, yet the reads make up the majority in the higher failed read length bins. In our regular production pipeline, we often circumvent this problem by using a combination of a 3' primer to resolve sequence immediately following the poly A coupled with an oligo(dT)_23_N (N = A, G, C) anchored primer to resolve the 3' ends of cDNA inserts.

## Conclusions

The FMA pipeline described here was tailored to our high-throughput 384-well automated sequencing pipeline but many of the components in this platform are shared within the high-throughput sequencing community such as the alkaline lysis procedure to prepare and purify DNA, DNA sequencing using Big Dye chemistry, ethanol precipitation to clean up reaction products and equipment such as the Genetix QPIX, Beckman Coulter Biomek FX, and Applied Biosystems 3730 xl DNA analyzer. For this reason our FMA methods can be readily adapted to analyze other similar sequencing platforms. Extending our present study to other library types commonly sequenced in the high-throughput community, such as shotgun or Serial Analysis of Gene Expression (SAGE) should offer further information regarding template specific failures. This failure mode analysis provides information on distinguishing between process and template-related attributes that may lead to downstream failed sequence. It can therefore be a useful tool used to audit overall sequencing procedures and identify key problematic steps in a process pipeline. Making proper adjustments to the pipeline based on the results will likely result in increased efficiency, enhanced data quality, and decreased cost.

## Methods

Samples are processed in duplicate, with quality assurance and observational checks to account for status of each read after every procedure leading to the final sequence read. These recorded observations facilitate downstream systematic analysis of failed sequence data. The regular core production pipeline was not altered for this study, as the purpose is to assess sequence failure modes under ordinary conditions in a high-throughput sequencing environment. An overview of the observational checkpoints within the FMA pipeline is outlined in Figure 1. Template DNA is considered present in all wells up to the completion of DNA sequencing unless absence is indicated by no growth of bacterial culture or blank lane on gel. Once cycle sequencing has completed, the reaction products are precipitated, pelleted, then washed. As it is very difficult to qualitatively asses the presence of DNA accurately after the precipitation procedures other than by sequencing, we draw conclusions regarding loss of sequencing reaction products during precipitation by a process of elimination. Any failed reads that result from a process-related loss of DNA and have no prior observations indicating absence of DNA are attributed to the precipitation procedure.

### Transformation and colony picking

One microliter of ligation mix from each of two *Populus trichocarpa *cDNA libraries were transformed by electroporation into 40 μl of *E. coli *DH10B T1 resistant cells (Invitrogen). Transformed cells were recovered using 1 mL of SOC medium (prepared in house) and plated onto 22 cm × 22 cm agar plates (Genetix) containing 2xYT agar and 100 μg/μl Ampicillin. Agar plates were incubated overnight at 37°C for 14 hours. Bacterial colonies were picked from the agar plates and arrayed into 384-well microtiter plates (Genetix) containing 60 μl of 2xYT medium + 7.5% glycerol (made in house) using the Genetix QPIX automated colony picker (Genetix). A total of six 384-well microtiter plates, three plates for each of the two cDNA libraries, were picked. The plates were incubated overnight at 37°C for 16 hours then each microtiter plate was inspected for wells that contained no growth. The positions of all failed cultures were recorded. To further verify growth in each well after the incubation period, a disposable 384-well replicator was used to stamp bacterial culture from each 384-well microtiter plate onto a new 22 cm × 22 cm agar plate containing 2xYT agar and 100 μg/ul ampicillin. Agar plates were incubated overnight at 37°C for 14 hours then inspected the next day for colonies from every well. The positions of all failed growths were recorded.

### 384-well culturing and DNA purification of plasmid clones

Two microliters of bacterial culture was transferred from the 384-well microtiter plate into a 240 μl 384-well deep well diamond plate (Axygen) containing 60 μl of 2xYT medium and 100 μg/ml ampicillin using a 384-well slotted inoculator (V&P Scientific). This was done in duplicate to create two sets of six 384-well deep well inoculated diamond plates. Inoculated plates were sealed with AirPore™ tape (Qiagen) and placed into a 37°C shaking incubator (New Brunswick Scientific C25 Incubator Shaker) at 350 rpm for 18 hours. After the incubation period, cultures were removed and each plate inspected for growth and contamination. All failed cultures were recorded. Cultures from both replicates were then placed onto separate multi-tube floor vortexers (VWR) at maximum speed for approximately 5 minutes until all cells were resuspended. Cultures were stored at 4°C until ready for DNA preparation. DNA was prepared using alkaline lysis [3] with the following modifications that have been implemented for the standard GSC template production pipeline. Culture blocks from both replicates were removed from the 4°C refrigerator and mixed using a multi-tube floor vortex (VWR) for 5 minutes at maximum speed (or until all cells appeared resuspended). A Titertek MapC2 liquid handling device was used to dispense 60 μl of Lysis Buffer (Qiagen Buffer P2). After 5 minutes of lysis, 60 μl of Neutralization Buffer (Qiagen Buffer P3) was added. Plates were tape sealed (Edge biosystems clear tape) and mixed on a multi-tube vortex at maximum speed for 2 minutes prior to centrifugation at 4250 × g for 45 minutes in a Jouan KR422 centrifuge. 120 μl of lysate were transferred from pelleted culture blocks into 240 μl 384-well deep well diamond plates containing 90 μl per well 100% isopropanol using a 384-well Hydra pipetting instrument (Robbins Scientific). Destination plates were sealed (Edge biosystems clear tape) and mixed by inversion, followed by centrifugation at 2830 × g for 15 minutes in an Eppendorf 5810R centrifuge (Brinkmann Instruments). After centrifugation the isopropanol was decanted, the DNA pellet washed with 50 μl 80% ethanol using a Robbins 384-well Hydra, and the plates left to dry upright for three hours on the benchtop. DNA pellets were resuspended in 10 mM Tris-HCl, pH = 8 containing 10 μg/ml RNase A (Qiagen) and mixed for 1 minute at maximum speed on a multi-tube vortexer. Plates were briefly centrifuged at low speed, stored at 4°C overnight, then transferred to a -20°C freezer until required for DNA evaluation and sequencing reactions.

### DNA evaluation

DNA preparations were evaluated by agarose gel electrophoresis. A 1.5 μl aliquot of purified DNA was combined with 1.5 μl of bromophenol blue loading buffer (0.21% bromophenol blue; 12.5% ficoll) and 2 μl was loaded onto a 1.2% agarose gel. Samples were loaded using a 12-channel loader (Hamilton) beside 3 ng of 1 kb plus DNA marker (Invitrogen). Gels were run at 120 volts for 90 minutes in TAE (Tris/Acetate/EDTA) buffer followed by staining for 35 minutes in SybrGreen Nucleic Acid stain (Cambrex). Gels were scanned using a Fluorimager 595 (Molecular Dyanmics) scanner. The image was visually examined for genomic DNA, as well as presence and quality of DNA. All empty lanes and observations were recorded and entered into the FMA database.

### DNA sequencing

DNA Sequencing reactions were assembled in 384-well clear optical reaction plates (Applied Biosystems) using a Biomek FX workstation (Beckman-Coulter). In each 5 μl reaction (total volume) the following were added: 3 μl of purified plasmid DNA (~45 ng/μl), 0.26 μl of sequencing primer (5 pmol/μl, Invitrogen), 0.43 μl of 5X reaction buffer (Applied Biosystems Big Dye Terminator 5X Sequencing Buffer), 0.77 μl of Ultrapure water (Gibco), and 0.54 μl of BigDye v.3.1 ready reaction mix (Applied Biosystems). Each well of the reaction plate was visually inspected for appropriate volumes after both reaction mix and DNA addition. Volumes were manually adjusted using a single channel pipet (Gilson) where required and all observations were recorded. Sequence data were obtained using universal M13 Forward (5'-TGTAAAACGACGGCCAGT-3') and M13 Reverse (5'-CAGGAAACAGCTATGAC-3') primers on each set of replicate plates. Thermal cycling was performed on PTC-225 thermal cyclers (MJ Research) with parameters of 35 cycles at 96°C for 10 seconds, 52°C for 5 seconds using M13 Forward primer or 43°C for 5 seconds using M13 Reverse, 60°C for 3 minutes, followed by incubation at 4°C. Reaction products were precipitated by adding 2 μl of 125 mM EDTA (pH8) and 18 μl of 95% ethanol per well followed by centrifugationat 2750 × g for 30 minutes in an Eppendorf 5810R centrifuge. The EDTA/ethanol was immediately decanted and reaction products washed with 70% ethanol. The 384-well cycle plates were allowed to dry inverted for 15 minutes. Samples were resuspended in 10 μl of Ultrapure water and analyzed using a 3730XL DNA analyzer (Applied Biosystems). The performance of each capillary on the four DNA analyzers used in this experiment were validated using one 384-well control plate for each instrument. Each 384-well plate contained our in-house control standard, a full-length human cDNA clone obtained from the I.M.A.G.E. Consortium (I.M.A.G.E. ID #3609158, Lawrence Livermore National Laboratories). Blocked capillaries were recorded into the FMA database and traces originating from these capillaries flagged. Sequence data were evaluated using PHRED software [2] (v.0.020425.c) and the chromatograms were viewed using a java applet based on 'ted' [4] – a publicly available trace file viewer. A relational database "FMAdb" was created using MySQL for flexible querying of results. The FMAdb was populated with sequence data, plus process observations such as absence of bacterial colonies and no-grows, trace evaluations and information, as well as equipment and sequence run details.

## Authors' contributions

GSY was responsible for design and execution of this study, plus analysis of data and drafting of the manuscript. RAH conceived of the study and directed the analysis. JMS and SAB generated the data described in the study and participated in study design. DS developed several of the key protocols used in this study. MB provided quality assurance of the experimental procedures and participated in study design. MAM participated in the establishment of the production pipeline and read the manuscript and provided comments.
